# Leveraging an mRNA Platform for the Development of Vaccines Against Egg Allergy

**DOI:** 10.3390/vaccines13050448

**Published:** 2025-04-24

**Authors:** Xianyu Shao, Lijing Liu, Changzhen Weng, Kun Guo, Zhutao Lu, Lulu Huang, Zhenhua Di, Yixuan Guo, Guorong Di, Renmei Qiao, Jingyi Wang, Yong Yang, Shiyu Sun, Shentian Zhuang, Ang Lin

**Affiliations:** 1 Center for Infectious Medicine and Vaccine Research, School of Basic Medicine and Clinical Pharmacy, China Pharmaceutical University, Nanjing 211198, China; 2 Institute of Translational Medicine, China Pharmaceutical University, Nanjing 211198, China; 3 Innovation Center for Nucleic Acid Medicine, Institute for Innovative Drug Development and Life Sciences, Wuxi 214000, China; 4 Center for New Drug Safety Evaluation and Research, China Pharmaceutical University, Nanjing 211198, China; 5 School of Pharmaceutical Sciences, Institute of Immunopharmaceutical Sciences, Shandong University, Jinan 250012, China

**Keywords:** mRNA technology, food allergy, egg allergy, anaphylaxis, preventive effect

## Abstract

**Background:** Food allergy (FA) poses a major global health issue due to the increasing prevalence and lack of effective prevention strategies. Allergen-specific immunotherapy (AIT) has emerged as a disease-modifying therapy for FA. However, due to long-term treatment duration and unexpected adverse reactions, only a minority of patients benefit from AIT. Therefore, effective prophylactic interventions are urgently needed for FA patients. **Methods:** In this proof-of-concept study, using a well-established mRNA platform, we developed mRNA vaccine candidates encoding for the major egg white allergen Gal d2 and comprehensively evaluated their prophylactic efficacy against anaphylaxis in a Gal d2-induced allergic mouse model. **Results:** Two vaccine formulations, Gal d2 mRNA vaccine and Gal d2-IL-10 mRNA vaccine, both demonstrated potent ability in inducing allergen-specific IgG and Th1-type T cells. Importantly, the two vaccine formulations showed promise in preventing the onset of allergic disease, which is indicated by prevention of body temperature decline during anaphylaxis. **Conclusions:** We provided preliminary proof-of-concept evidence showing that the mRNA platform is unique and holds promise for the development of anti-allergy vaccines. This is largely attributed to the capacities of mRNA vaccines in eliciting an allergen-blocking antibody, shifting Th2 towards Th1 immunity, as well as in generating peripheral tolerance. However, further investigations are required to better understand the mode of action.

## 1. Introduction

Allergic diseases, also referred to as type I hypersensitivity reactions, have posed a major global health burden due to the rising prevalence and lack of effective prophylactic strategies. Among various types of allergic conditions, food allergy (FA) represents a critical category and affects about 8% of children and 10% of adults in developed countries [1]. In the Chinese population, prevalence of FA has been increasing rapidly and was reported to range from 4.0% to 8.2% according to a meta-analysis [2]. Currently, strict self-avoidance of specific food is commonly used as a strategy by the majority of FA patients, but accidental allergen exposure still occurs frequently, which requires immediate rescue medication, otherwise a life-threatening systemic allergic reaction known as anaphylaxis may appear in some severe cases. During the past decades, allergen-specific immunotherapy (AIT) has been used as a disease-modifying therapy for food allergic conditions, including peanut allergy, wheat allergy, etc., which is practiced by exposing the patients to gradually increasing amounts of the allergen in controlled dosing to induce tolerance [3]. However, due to the long-term treatment duration, poor patient compliance, and unexpected adverse reactions, only a minority of patients benefit from AIT therapy. Therefore, effective prophylactic interventions are largely needed for FA patients.

Allergic disorders are typically characterized by aberrant generation of Th2-polarized immune responses indicated by the induction of allergen-specific IgE and Th2-type cytokines (IL-14, IL-5, IL-13, etc.) secreting T cells that collaboratively lead to activation of effector cells such as basophils and mast cells, which play a critical role in mediating and exaggerating allergic reactions [4]. Taking advantage of the principles of the immune system, considerable attempts have been made to develop anti-allergy vaccines to prevent FA incidence by inducing allergen-blocking antibodies competing with IgE and modulating or even converting allergen-specific Th2 immunity to a Th1 phenotype [5]. Using defined food allergens as immunogens, vaccine modalities based on a virus-like particle (VLP) platform, nucleic acid platform, viral-vectored platform, and adjuvanted protein-based subunit vaccine platform have been tested and showed promise in mitigating allergic response to food allergens [6,7,8,9,10]. While compared with traditional vaccine types, the mRNA vaccine type is superior at inducing stronger Th1-type T-cell responses and a higher magnitude of Ab responses. Moreover, an mRNA vaccine formulated using a lipid–nanoparticle (LNP) delivery system was reported to induce a regulatory T-cell (Treg) response, contributing to immune tolerance [11,12], which provides an additional advantage to mRNA technology for anti-allergy vaccine development. In this proof-of-concept study, using a well-established mRNA platform [13,14,15], we developed mRNA vaccine candidates encoding for the major egg white allergen Gal d2 (also known as ovalbumin) and comprehensively evaluated the prophylactic efficacy against anaphylaxis in a Gal d2-induced allergic mouse model.

## 2. Materials and Methods

A detailed description of the materials and methods used in this study can be found in the Supplementary Materials.

### 2.1. Ethics and Animals

BALB/c mice (female, 6-week-old) were purchased from GemPharmatech Co., Ltd. (Nanjing, China) and randomly allocated to different groups. Mice were kept in specific pathogen-free (SPF) condition at the Center for New Drug Safety Evaluation and Research at China Pharmaceutical University. All animal experiments were performed in accordance with the Guidelines for the Care and Use of Laboratory Animals and the Ethical Committee of China Pharmaceutical University using protocols approved by the Institutional Animal Care and Use Committee of China Pharmaceutical University (approval number: VR-B2301P090).

### 2.2. mRNA Vaccine Preparation

mRNAs encoding for Gal d2 or Gal d2-IL-10 were codon-optimized and synthesized by T7 polymerase-mediated in vitro transcription (IVT) using a linearized DNA template (pUC57-GW-Kan) containing 5′ untranslated regions (UTRs), 3′ UTRs and a 120 nt poly-A tail. Regarding the design of the Gal d2-IL-10 mRNA construct, a self-cleaving T2A peptide (GSGEGRGSLLTCGDVEENPGP) was inserted to bridge the Gal d2-mRNA and IL-10-mRNA. During the IVT procedure, N1-Methyl-pseudouridine (Synthgene, Nanjing, China) was used to modify the mRNAs, and the mRNAs were capped using Cap 1 Analogue Reagent (Synthgene, Nanjing, China). Subsequently, IVT products were purified using Monarch RNA purification columns (NEW ENGLAND BioLabs Inc., Ipswich, MA, USA) and resuspended in TE buffer. The lipid components were dissolved in ethanol at molar ratios of 50:10:38.5:1.5 (SM102:DSPC:cholesterol:DMG-PEG2000, purchased from SINPOEC, Xiamen, China). The mRNAs were dissolved in 10 mM citrate buffer (pH 4.0) and then encapsulated into lipid nanoparticles (LNPs) at a volume ratio of 3:1 using a microfluidic-based device (INanoTML from Micro & Nano Biologics, Suzhou, China) at a flow rate of 12 mL/min. The formulations were diluted with PBS and ultrafiltrated using 50-kDa Amicon ultracentrifugal filters (MilliporeSigma, Burlington, MA, USA). The vaccine formulations were characterized by NanoBrook Omni ZetaPlus (Brookhaven Instruments, Nashua, NH, USA) for particle diameter, polymer dispersity index, and zeta potentials. No additional adjuvant was included in the vaccine formulation.

### 2.3. Measurement of the Gal d2-Specific Antibody Titer

96-well plates (Greiner Bio-One) were pre-coated with Gal d2 antigen (100 µg/mL) at 4 °C overnight. The plates were washed three times with PBS containing 0.075% Tween-20 (PBST) and blocked by 2% bovine serum albumin (BSA) dissolved in PBST at 30 °C for 1 h. For detection of the Gal d2-specific IgG, IgG1, and IgG2a sera samples serially diluted at two-fold (starting from 1:400) were added into the wells and incubated for 2 h at 30 °C. Then HRP-conjugated rabbit anti-mouse IgG antibodies (1:50,000 dilution, Abcam, Cambridge, UK), IgG1 antibodies (1:5000 dilution, Southern Biotech, Birmingham, AL, USA), or IgG2a antibodies (1:5000 dilution, Southern Biotech, Birmingham, AL, USA) were added and incubated for 1 h at 30 °C, respectively. For detection of the Gal d2-specific IgE titer, sera samples (1:10 diluted) were added to the wells and incubated for 2 h at 30 °C, followed by incubation with HRP-conjugated rabbit anti-mouse IgE antibodies (Southern Biotech, 1:5000 dilution) for 1 h at 30 °C. After washing steps, TMB substrate was used for development and the absorbance was measured at 450 nm using the SpectraMax^®^ Absorbance Reader (Molecular Devices, San Jose, CA, USA).

### 2.4. Enzyme-Linked Immunospot (ELISPOT) Assay

Frequencies of the Gal d2-specific IL-2, IFN-γ, or IL-10 secreting T cells were measured using commercial ELISpot kits purchased from MABTECH (Stockholm, Sweden) according to the instructions. In brief, murine splenocytes (0.2 million cells per well) were incubated with or without an overlapping peptide pool (Miltenyi, Bergisch Gladbach, Germany) at the concentration of 10 μg/mL for 24 h. Cells stimulated with S. aureus Enterotoxin Type B Toxoid (SEB, Creative Diagnostics, Shirley, NY, USA) were treated as the positive control. The primary antibody (detection antibody coupling biotin) was diluted with antibody diluent at the ratio of 1:1000 (final concentration: 1 μg/mL) and added to each well, and the plates were incubated for 2 h at room temperature. Following washing steps, secondary antibody (Streptavidin-ALP) was diluted with antibody diluent at 1:1000 ratio and added to each well, and the plates were then incubated for 1 h at room temperature. After washing, filtered BCIP/NBT-plus solution was added to each well for color development in the dark. Spots were counted using CTL-Immunospot S6 Analyzer (ImmunoSpot, Cleveland, OH, USA). Results were depicted as spot-forming cells (SFCs) per million stimulated cells.

### 2.5. Statistical Analysis

Statistical calculations were performed using GraphPad Prism v8.0. Statistical difference among three or more groups was analyzed by non-parametric one-way ANOVA (Kruskal–Wallis) test. A *p* value less than 0.05 was considered statistically significant (* *p* ≤ 0.05, ** *p* ≤ 0.01, *** *p* ≤ 0.001, **** *p* ≤ 0.0001).

## 3. Results

**Figure 1:**
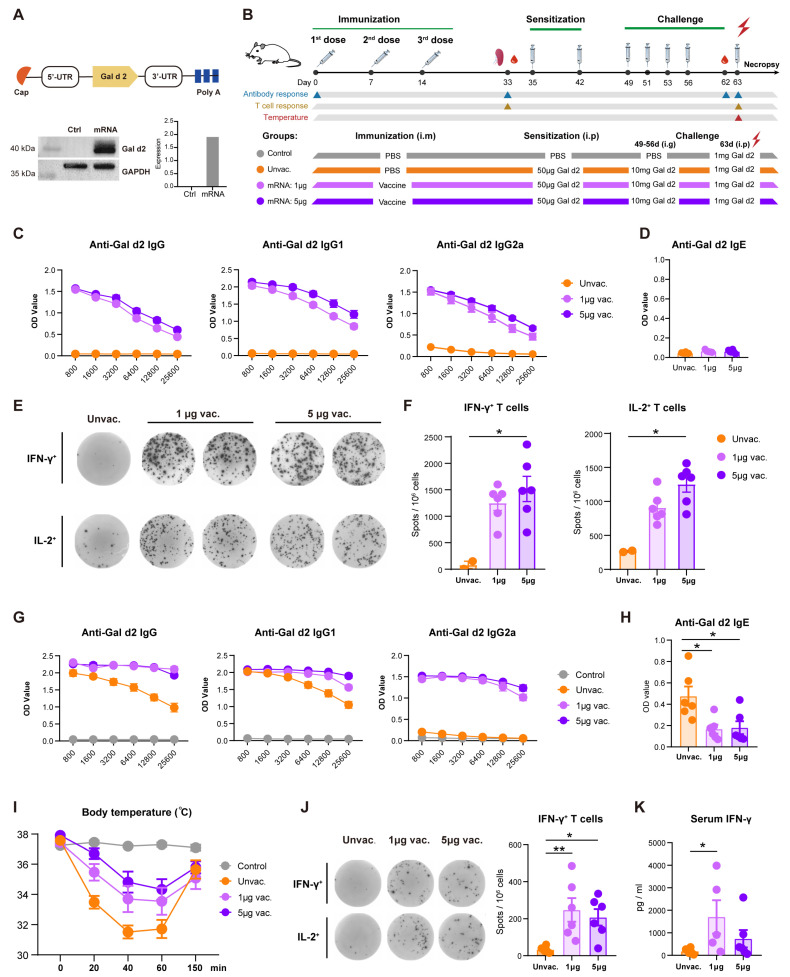
Immunogenicity and anti-allergy efficacy of the Gal d2 mRNA vaccine. (**A**) Construction of the Gal d2-mRNA and its translational efficiency in HEK-293T cells was assessed by western blot. (**B**) Experimental design. BALB/c mice (n = 12) were immunized three times at days 0, 7, and 14, followed by Gal d2 sensitization and i.g. challenge, consecutively. The final Gal d2 challenge was i.p. administered to induce anaphylaxis. (**C**,**D**) Gal d2-specific IgG, IgG1, IgG2a, and IgE in mice (n = 6) were measured at day 33. (**E**,**F**) Frequencies of the Gal d2-specific IFN-γ- or IL-2-producing T cells in spleens were measured by ELISpot assay. (**G**,**H**) At day 62 prior to the final allergen challenge, Gal d2-specific IgG, IgG1, IgG2a, and IgE in the mice (n = 6) were measured. (**I**) Following the final i.p. allergen challenge, rectal temperature of the mice was monitored. (**J**) Four hours post the final i.p. allergen challenge, levels of the Gal d2-specific IFN-γ or IL-2-producing T cells were quantified (n = 6) and the serum level of IFN-γ was measured (**K**). Non-parametric one-way ANOVA (Kruskal–Wallis) test was used for statistical analysis. * *p* ≤ 0.05, ** *p* ≤ 0.01.

Gal d2-mRNA was codon-optimized using a proprietary artificial intelligence-based algorithm and synthesized by an in-vitro transcription procedure with N1-methyl-pseudouridine (m1Ψ) incorporated, as previously reported [13], which demonstrated efficient protein expression following transfection into HEK-293T cells (Figure 1A). Vaccine formulation was prepared by packaging the mRNAs into an SM102-containing LNP system using a microfluidics-based procedure. To study the immunogenicity and anti-allergy effect of the vaccine, BALB/c mice were administered intramuscularly (i.m.) with three doses of vaccine (1 μg or 5 μg) at an interval of 7 days. Thereafter, mice were sensitized twice via intraperitoneal (i.p.) administration with Gal d2 followed by intragastric (i.g.) allergen challenge four times consecutively, and anaphylaxis was finally induced through i.p. injection with Gal d2 (Figure 1B). At day 33 prior to Gal d2 sensitization, mice immunized with Gal d2-mRNA vaccines produced robust levels of Gal d2-specific IgG and two IgG subclasses in a dose-dependent manner (Figure 1C). Anti-Gal d2 IgE was undetectable, therefore excluding the possibility that the mRNA vaccine might elicit an IgE response (Figure 1D). In addition, Gal d2-mRNA vaccination elicited strong Th1-type T cells secreting IFN-γ or IL-2, which is quite expected (Figure 1E,F). As a control group, unvaccinated mice showed no or very limited background levels of Ab or T-cell responses (Figure 1C–F).

Following the immunization procedure, animals underwent a series of allergen sensitizations and a challenge schedule to achieve allergic status to Gal d2. At day 62 prior to the final i.p. injection to induce anaphylaxis, levels of Gal d2-specific IgG and IgE were evaluated. Mice that had been immunized with vaccine maintained high levels of specific IgG, IgG1, and IgG2a, which were even at a higher magnitude than that detected at day 33 (Figure 1G). This can be explained by the continuous exposure to the antigen during the sensitization and challenge stages that boosted the memory B-cell pool. Interestingly, unvaccinated mice that had undergone the allergen sensitization and challenge regimen also produced a robust level of specific IgG that was exclusively composed of the IgG1 subclass; however, the Th1-prone antibody IgG2a was barely induced (Figure 1G). This suggested that Gal d2-allergic mice presented an aberrant allergen-specific Th2-type immune signature. Notably, unvaccinated Gal d2-allergic mice showed a significantly higher level of allergen-specific IgE than the two groups of mice that had been vaccinated (Figure 1H), which demonstrated the protective potential of Gal d2-mRNA vaccine since IgE is the key mediator of allergic response.

Further, anaphylaxis was induced via i.p. challenge with Gal d2, and rectal temperature, as the key indication of symptoms, was monitored. Mice in the control group (PBS treated) showed steady body temperature upon allergen challenge. However, unvaccinated allergic mice showed a sharp decline in temperature with a maximal drop observed at 40 min, and the temperature gradually recovered to normal state by 150 min (Figure 1I). In contrast, mice that were immunized with the Gal d2-mRNA vaccine showed a noticeably milder temperature drop, indicating a desired anti-allergy efficiency conferred by the mRNA vaccine, which was more prominently seen in the 5 μg dosing group. A dose-escalating experiment was also conducted to determine optimal vaccine doses. Neither the lower vaccine dose (0.2 μg) nor the higher vaccine dose (20 μg) demonstrated better protective efficacy than moderate doses (1 μg and 5 μg) against anaphylaxis-induced temperature drop (Supplementary Figure S1). We also evaluated the Gal d2-specific T-cell responses 4 h after the final allergen challenge. Compared with the unvaccinated group, immunized mice showed robust and higher frequencies of IFN-γ-producing T cells (Figure 1J) and higher levels of serum IFN-γ (Figure 1K). These collectively suggested that pre-vaccination with the Gal d2-mRNA vaccine was able to induce allergen-specific Th1-type T-cell immunity that counteracts with the generation of Th2-prone immunity, which may contribute to the manifestations of the anti-allergy effect.

**Figure 2:**
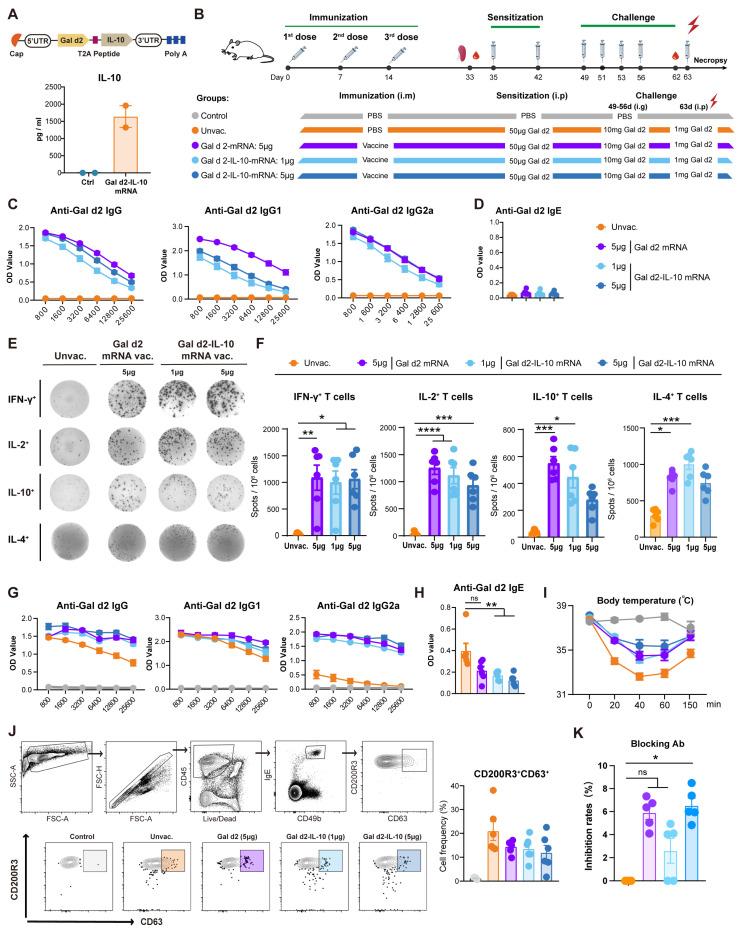
Immunogenicity and anti-allergy efficacy of the Gal d2-IL-10 mRNA vaccine. (**A**) Construction of the Gal d2-IL-10 mRNA (upper panel). Expression of IL-10 in the culture supernatant was determined by ELISA following mRNA transfection into HEK-293T cells (lower panel). (**B**) Experimental design. BALB/c mice (n = 12 each group) were immunized three times at days 0, 7, and 14, followed by Gal d2 sensitization and i.g. challenge, consecutively. The final Gal d2 challenge was i.p. administered to induce anaphylaxis. (**C**,**D**) Gal d2-specific IgG, IgG1, IgG2a, and IgE in the mice (n = 6 each group) were measured at day 33. (**E**,**F**) Frequencies of the Gal d2-specific IFN-γ-, IL-2-, IL-4-, or IL-10-producing T cells in spleens were measured by an ELISpot assay. (**G**,**H**) At day 62, prior to the final allergen challenge, Gal d2-specific IgG, IgG1, IgG2a, and IgE in the mice (n = 6) were measured. (**I**) Following the final i.p. allergen challenge, rectal temperature of the mice was monitored. (**J**) Four hours post the final i.p. allergen challenge, frequencies of activated basophils were measured by FACS. (**K**) The allergen-blocking capacity of the vaccine-induced antibodies was measured (n = 5 each group). Inhibition rate is shown. Non-parametric one-way ANOVA (Kruskal–Wallis test) was used for statistical analysis. * *p* ≤ 0.05, ** *p* ≤ 0.01, *** *p* ≤ 0.001, **** *p* ≤ 0.0001.

Given the above finding showing a promising anti-allergy effect of the Gal d2-mRNA vaccine, we further modified the vaccine modality by conjugating immune-suppressive cytokine IL-10 encoding mRNA with Gal d2-mRNA through a T2A self-cleaving peptide (Figure 2A). The T2A peptide can cause ribosomal skipping of the glycine–proline peptide bond at the C-terminus of the 2A element allowing for generation of the Gal d2 and IL-10 proteins separately. IL-10 is essential not only for the induction of peripheral tolerance to the allergen but also plays a critical role in the suppression of the IgE response [16]. Upon transfection into HEK-293T cells, the constructed Gal d2-IL-10 mRNA was efficiently translated with a robust level of IL-10 detected in the supernatant (Figure 2A). We further compared the immunogenicity and anti-allergy efficiency between the Gal d2-IL-10 mRNA vaccine and Gal d2 mRNA vaccine according to an identical experimental schedule as shown earlier (Figure 2B). In terms of antibody response, triple doses of 5 μg of the Gal d2-IL-10 mRNA vaccine induced a comparable level of IgG and IgG2a to the Gal d2 mRNA vaccine, while Th2-prone antibody IgG1 was elicited at a lower level by the Gal d2-IL-10 mRNA vaccine (Figure 2C). Again, anti-Gal d2 IgE was not elicited in all vaccine groups (Figure 2D). Moreover, robust levels of antigen-specific IFN-γ-, IL-2-, or IL-4-producing T cells were induced by the two vaccine formulations with no difference observed. While noticeably and interestingly, IL-10-secreting T cells were generated in all vaccine groups, which was even more robust in the Gal d2 mRNA vaccine group (Figure 2E,F). These vaccine-elicited IL-10^+^ T cells are likely functional in mitigating the allergic response, and the in-depth phenotyping and characterization would merit further investigation.

Following the sensitization and challenge schedule and prior to the final challenge to induce anaphylaxis, IgG, IgG1, and IgG2a titers were further boosted in all vaccine groups (Figure 2G), which was in line with the earlier finding (Figure 1G). The anti-Gal d2 IgE titer was significantly reduced in vaccinated animals but showed no difference between the two vaccine formulations (Figure 2H). Following the final allergen challenge, rectal temperature was monitored, and all vaccinated mice were protected from severe temperature decline during anaphylaxis when compared with the unvaccinated group. Of note, the Gal d2-IL-10 mRNA vaccine showed a comparable efficiency in alleviating allergic response to the Gal d2 mRNA vaccine (Figure 2I). Four hours after the allergen challenge, we evaluated the frequencies of activated basophils (IgE^+^CD200R3^+^CD63^+^) in the spleens, which are major effector cells mediating allergic reaction. This specific cell population was barely detected in the control PBS-treated mice but was present at a high level in the unvaccinated allergic mice. In contrast, vaccinated mice showed relatively lower frequencies of activated basophils with no clear difference between the two vaccine formulations (Figure 2J). Moreover, to assess whether mRNA vaccination shifted the allergen-specific Th2-type response to the Th1 phenotype, serum IFN-γ and IL-4 levels were quantified four hours post the final allergen challenge. Compared with the unvaccinated mice undergoing allergen challenge, the majority of vaccinated mice showed increased levels of IFN-γ, while no difference in serum IL-4 level was observed between vaccinated and unvaccinated animals (Supplementary Figure S2).

Collectively, the Gal d2 mRNA and optimized Gal d2-IL-10 mRNA vaccines demonstrated comparable ability in preventing the onset of allergic disease. With regards to the potential mechanisms, induction of a strong Th1-type immunity counteracting with Th2-type allergic immunity (Figure 1E and Figure 2E) and the generation of regulatory IL-10^+^ T cells (Figure 2E) may play a critical role. In addition, the mRNA vaccine-elicited IgG could function as blocking antibody competing with IgE for binding to the allergen. This was supported by competitive ELISA experiments showing that vaccine-elicited antibodies were able to block IgE-allergen interaction (Figure 2K).

## 4. Discussion

Growing evidence has recently suggested that the mRNA platform is versatile and holds promise for the development of vaccines or therapeutics for non-infectious diseases, such as autoimmune disease and cancer, as well as many other inflammatory disorders. With the increasing understanding of mRNA technology and the pathogenesis of allergic diseases, we supposed that the mRNA platform might be superior to other platforms in anti-allergy vaccine development. To this end, we performed this proof-of-concept study by developing mRNA vaccine candidates against one type of highly prevalent food allergic disease, egg allergy, and comprehensively evaluated their prophylactic efficacy against anaphylaxis in a Gal d2-induced allergic mouse model.

Our study showed that both the Gal d2 mRNA vaccine and the optimized Gal d2-IL-10 mRNA vaccine could elicit robust levels of specific IgG, which can function by blocking allergen-IgE interaction. Considering that allergic reactions occur when mast cells and basophils are activated through cross-linking of membrane-bound IgE during allergen exposure, the vaccine-elicited non-IgE antibodies could help to mitigate the onset of allergic response. We also noticed that the Gal d2-mRNA vaccine induced higher levels of specific IgG than the Gal d2-IL-10 mRNA vaccine when administered at an equivalent dose. However, the blocking effect of vaccine-elicited IgG was slightly more prominent in the Gal d2-IL-10 mRNA vaccine group. Considering that IL-10 can enhance the survival, proliferation, and differentiation of B cells, it remains to be answered whether IgG induced by the two vaccine formulations differ in their intrinsic ability, such as Ab avidity and clonality that may affect allergen binding.

Moreover, IFN-γ- or IL-2-secreting T cells are robustly induced through mRNA vaccination. This typical Th1-biased T-cell immunity could be utilized for modulating or even converting allergic Th2 reactions, which was also reported by previous studies showing that DNA vaccine-induced IFN-γ^+^ T-cell responses are associated with protection against allergy [17]. Our study indicated that upon anaphylaxis, vaccinated mice had higher levels of serum IFN-γ than unvaccinated mice, which suggested that pre-treatment with mRNA vaccines could partially shift allergen-specific Th2 T-cell response to the Th1 phenotype. In addition, IL-10-secreting T cells were induced by both the mRNA vaccine formulations. However, it remains largely unclear in terms of the role of these vaccine-elicited IL-10^+^ T cells in prevention of the allergic response. While it should be noted that previous studies have demonstrated that mRNA vaccines formulated with a specific LNP system could generate Treg responses [11,12], this unique property of the mRNA vaccine formulation could be further explored and harnessed for development of mRNA therapeutics for allergy or other autoimmune diseases.

There are some weaknesses of our study that await to be further addressed. One major point is that the therapeutic potential of mRNA vaccines in treating existing food allergy remains unknown. In addition, some immune assays used in our study could be further optimized. For example, to avoid the potential blocking effect by IgG, it would be more precise to use IgG-depleted sera or an IgE-capturing assay for detection of allergen-specific IgE. Basophil activation was only assessed in spleen tissue, which should also be studied in other tissues, for example peripheral blood and lymph nodes. Apart from these, in-depth investigations would be required to gain deep mechanistic insights into the preventive efficacy of the mRNA vaccine that could provide guidance for rational anti-allergy vaccine development.

## 5. Conclusions

In this brief report, we provided preliminary evidence showing that the mRNA platform is unique and holds promise for the development of anti-allergy vaccines. This is largely attributed to the versatilities of the mRNA vaccine in eliciting an allergen-blocking antibody, shifting Th2 towards Th1 immunity, as well as in generating peripheral tolerance.

## Data Availability

All data are available upon reasonable request to the corresponding authors.

## Supplementary Materials

The following supporting information can be downloaded at: https://www.mdpi.com/article/10.3390/vaccines13050448/s1, Figure S1: Dose-escalating experiment for evaluation of Gal d2-mRNA vaccine efficacy; Figure S2: Levels of serum IFN-γ and IL-4; Figure S3. Raw pictures of the western blot result shown in Figure 1.
