# Supplementary material for: Leveraging an mRNA Platform for the Development of Vaccines Against Egg Allergy
Source: Vaccines (Basel). 2025 Apr 24;13(5):448. doi: 10.3390/vaccines13050448 (PMC12116152; doi:10.3390/vaccines13050448)
Supplement: Supplementary file 1 [file vaccines-13-00448-s001.zip › vaccines-3581442-supplementary.pdf]

Supplementary Materials for

Leveraging an mRNA Platform for the Development of Vaccines Against Egg Allergy

Xianyu Shao [1,2,3,†], Lijing Liu [1,2,3,†], Changzhen Weng [1,2], Kun Guo [1,2,3], Zhutao Lu [1,2], Lulu Huang [1,2], Zhenhua Di [1,2], Yixuan Guo [1,2], Guorong Di [1], Renmei Qiao [1], Jingyi Wang [1,2], Yong Yang [1,2,4], Shiyu Sun [1,2], Shentian Zhuang [1,2,*] and Ang Lin [1,2,3,5,*]

1 Center for Infectious Medicine and Vaccine Research, School of Basic Medicine and Clinical Pharmacy, China Pharmaceutical University, Nanjing 211198, China
2 Institute of Translational Medicine, China Pharmaceutical University, Nanjing 211198, China
3 Innovation Center for Nucleic Acid Medicine, Institute for Innovative Drug Development and Life Sciences, Wuxi 214000, China
4 Center for New Drug Safety Evaluation and Research, China Pharmaceutical University, Nanjing 211198, China
5 School of Pharmaceutical Sciences, Institute of Immunopharmaceutical Sciences, Shandong University, Jinan 250012, China
* Correspondence: shentianzhuang@cpu.edu.cn (S.Z.); anglin@cpu.edu.cn (A.L.)
† These authors contributed equally to this work.

Lead contact:

Ang Lin, anglin@cpu.edu.cn

Supplementary Materials and Methods

Cell culture

HEK-293T cells were purchased from Cell Resource Center, Shanghai Institutes for Biological Sciences, Chinese Academy of Sciences and cultured in DMEM (BIOIND, Israel) supplemented with 10% fetal bovine serum (FBS, BIOIND, Israel) and 1% penicillin-streptomycin (NCM Biotech, China). All cells were maintained at 37 °C and in a 5% CO_2 atmosphere.

Immunization and sampling

To evaluate the immunogenicity and preventive effect of vaccine, three doses of Gal d2 mRNA or Gal d2-IL-10 mRNA vaccines were i.m. administered to BALB/c mice (start with n=12 per group) at an interval of 7 days. Sera samples (n=6 per group) were collected longitudinally at indicated time points for analyses of antibody responses. Following immunization schedule, mice from indicated groups were sensitized by i.p. injection of 50 μg of Gal d2 (Sigma Aldrich) mixed with 2 mg of alum adjuvant (Thermo Scientific) per dose at day 35 and 42, followed by i.g. challenge with 10 mg of OVA at day 49, 51, 53 and 56. One week post the final i.g. challenge, mice were i.p. challenged with 1 mg of Gal d2 to induce anaphylaxis. Rectal temperature at indicated time points was recorded with a rectal thermometer. Four hours post the final i.p. challenge, animals were sacrificed, and spleens were processed to obtain single cell suspension. Overview of the experimental schedule is displayed in Figure 1b and Figure 2b.

Preparation of single cell suspension

Spleen tissues were grounded gently and filtered through a 70-μm sterile cell strainer. Cells were resuspended in PBS and centrifuged at 400 g for 10 min. To lyse red blood cells (RBC), cell pellets were resuspended with RBC lysis buffer (Solarbio) for 5 min at 4 °C. Following this, 1× PBS was added to terminate the lysis procedure and cells were then washed at 400 g for 10 min to obtain splenic MNCs. Thereafter, cells were

re-suspended in RPMI-1640 medium containing 10% FBS (BIOIND) and 1% penicillin-streptomycin (NCM Biotech) for subsequent in-vitro experiments.

Western blot assay

HEK-293T cells were transfected with mRNAs using jetMESSENGER® transfection reagent according to the instruction. Upon 24 hours of incubation, cells were collected and lysed, followed by centrifugation (12,000 rpm, 15 min, 4 °C) for protein extraction. Protein concentration was determined by BCA Protein Quantification Kit-BOX 2 (Vazyme). 20 μg of proteins were loaded onto a 10% SDS-polyacrylamide gel for SDS-PAGE electrophoresis. Proteins were then transferred onto PVDF membranes and were blocked with 5% milk for 2 hours at room temperature (RT). The membrane was then washed with TBS containing 0.075% Tween-20 (TBST) for 3 times and incubated with 1: 10000 diluted anti-Gal d2 antibody (Proteintech, CloneNo.1D3D5) at 4 °C overnight. After washing steps, the membrane was incubated with 1: 10000 diluted HRP-conjugated goat anti-mouse IgG (Fdbio science) for 2 h. The membrane was washed, and spots were visualized using Amersham ECL Prime Western Blotting Detection Reagent (GE Healthcare).

Enzyme Linked Immunosorbent Assay (ELISA)

Level of serum IFN-γ or IL-10 in the culture supernatants were measured using commercial ELISA kits purchased from MultiSciences Biotech and Elabscience Biotec, respectively. Measurements were performed according to the manuals. Tecan sunrise Microplate reader was used for the detection of the absorbance at 450 nm.

Competitive ELISA assay

To assess the allergen-blocking capacity of vaccine-induced antibody, sera samples collected from mice that had been immunized with three doses of mRNA vaccines at an interval of 7 days were used. 96-well plates were pre-coated with 100 μg/mL Gal d2 and incubated overnight at 4 °C. Upon washing with PBST and blocked by 2% BSA at 37 °C for 2 h, sera collected from unvaccinated naive mice or vaccinated mice

(n=5) were heat-inactivated at 56 °C for 2 hours and then added into the plates for incubation at 4 °C for 1 hour. PBS was added into additional wells and served as negative control. Following washing steps, sera samples containing Gal d2-specific IgE that was collected from Gal d2-allergic mice were 1: 10 diluted and added to the wells for overnight incubation at 4 °C. After washing, HRP-conjugated rabbit anti-mouse IgE antibodies (1:5000 dilution) were added for incubation at 30 °C for 1 hour. After washing, TMB substrate was used for development, and the absorbance was measured at 450 nm. The inhibition rate (%) was calculated as follows: $100 - [(OD_i / OD_{PBS}) \times 100]$, where OD_i represents the optical density value of test mouse sera from mRNA vaccine immunized mice or from naïve mice, and OD_{PBS} denotes the optical density of the wells with PBS added.

Evaluation of basophil activation

Frequencies of activated basophils (identified as $IgE^+CD200R3^+CD63^+$ cells) were quantified using flow cytometric assay. In brief, 2×10^6 murine splenocytes were washed with PBS and stained with LIVE/DEAD™ Fixable Aqua Dead Cell Stain Kit (Thermo) for 5 minutes and then incubated with antibody cocktails and Fc receptor blocking reagent (Miltenyi) for 20 minutes at 4°C in dark. Antibodies used in this analysis include anti-mouse CD45-Pacific Blue™ (clone: 30-F11, Biolegend), anti-mouse IgE-PE (clone: RME-1, Biolegend), anti-mouse CD49b-Percp-Cy5.5 (clone: HMα2, Biolegend), anti-mouse CD200R3-AF647 (clone: Ba13, Invitrogen), anti-mouse CD63-AF700 (clone: NVG-2, Biolegend). Flow cytometric analysis was performed on BD FACSCelesta. Data were analyzed with FlowJo software (version 10.8.1).

Supplementary Figure S1

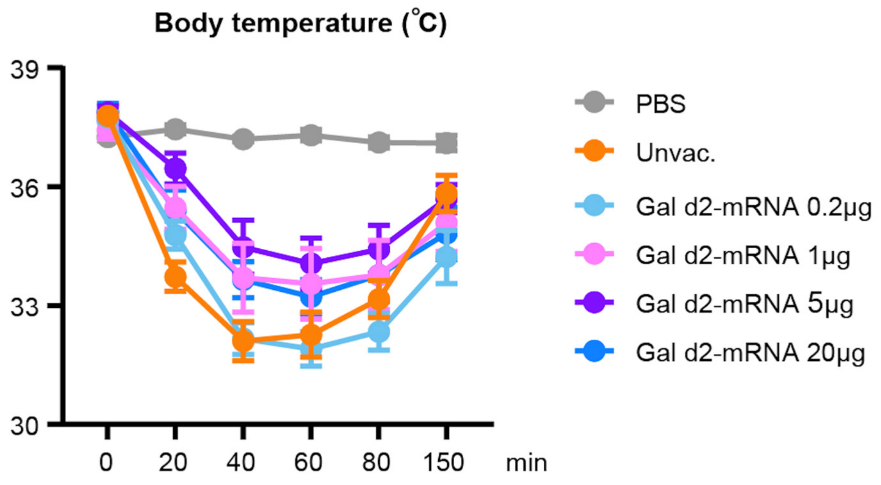

Figure S1. Dose-escalating experiment for evaluation of Gal d2-mRNA vaccine efficacy. BALB/c mice were immunized with escalating doses of Gal d2 mRNA vaccines at day 0, 7, and 14, followed by Gal d2 sensitization and i.g. challenge consecutively. Following final i.p. allergen challenge, rectal temperature of mice was monitored.

Supplementary Figure S2

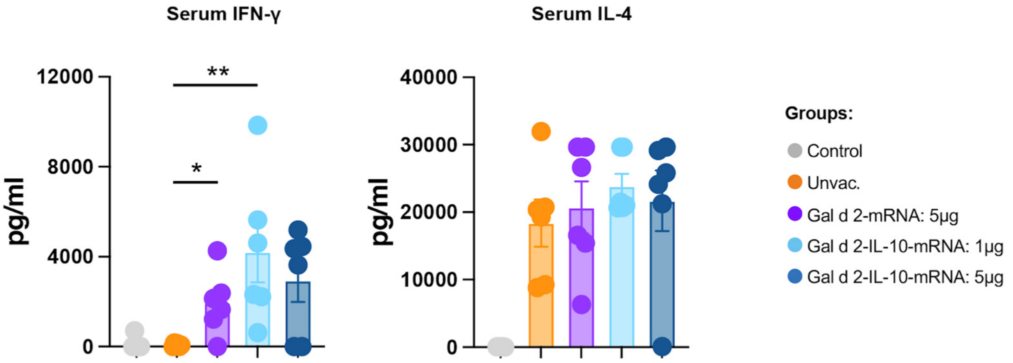

Figure S2. Levels of serum IFN-γ and IL-4. BALB/c mice (n=6 each group) were immunized three times at day 0, 7, and 14, followed by Gal d2 sensitization and i.g. challenge consecutively. Final Gal d2 challenge was i.p. administered to induce anaphylaxis. Four hours post the final i.p. allergen challenge, serum IFN-γ and IL-4 levels were measured by ELISA. Non-parametric one-way ANOVA (Kruskal-Wallis) test was used for statistical analysis. $^*p \leq 0.05$, $^{**}p \leq 0.01$.

Supplementary Figure S3

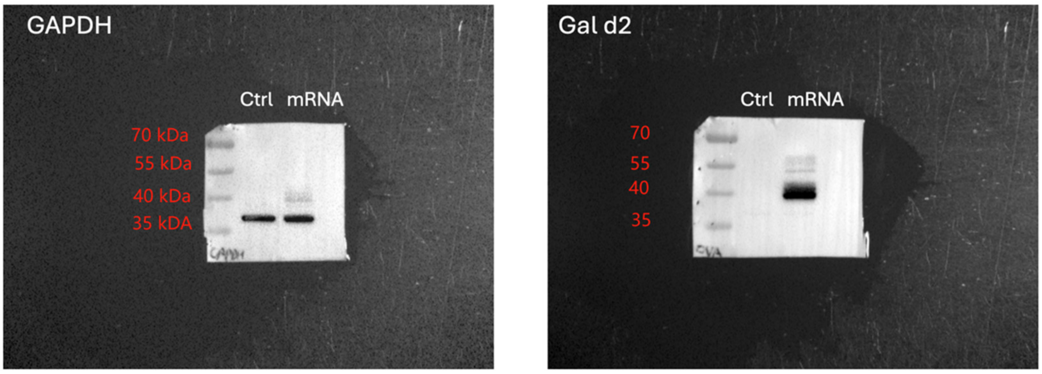

Figure S3. Raw pictures of the western blot result shown in Figure 1.
